# Examining the association between meal context and diet quality: an observational study of meal context in older adults

**DOI:** 10.1186/s12966-021-01122-x

**Published:** 2021-05-20

**Authors:** Marissa M. Shams-White, Robert W. Korycinski, Kevin W. Dodd, Brian Barrett, Stephanie Jacobs, Amy F. Subar, Yikyung Park, Heather R. Bowles

**Affiliations:** 1 Division of Cancer Control and Population Sciences, Epidemiology and Genomics Research Program, National Cancer Institute, 9609 Medical Center Drive, Bethesda, MD 20892 USA; 2 Division of Cancer Prevention, National Cancer Institute, Bethesda, MD 20892 USA; 3 Information Management Services, Inc., Rockville, MD 20850 USA; 4 Division of Public Health Sciences, Department of Surgery, Washington University School of Medicine, St. Louis, MO USA

**Keywords:** ASA24, Food away from home, Food environment, Healthy eating index, Meal location, Screen time, Social support

## Abstract

**Background:**

Though a healthy diet is widely associated with reduced risks for chronic disease and mortality, older adults in the U.S. on average do not meet dietary recommendations. Given that few studies have examined the association between meal context on older adult diet quality, the aims of this study were (1) to compare the dietary quality of foods consumed in different meal contexts, as measured by the Healthy Eating Index 2015 (HEI-2015): meal location, the presence of others, and the use of electronic screens; and (2) to examine which components of the HEI-2015 drove differences in HEI-2015 total scores by meal context.

**Methods:**

Interactive Diet and Activity Tracking in AARP study participants (50–74 years) completed the Automated Self-Administered 24-h Dietary Assessment tool (ASA24, version 2011) that included foods and beverages consumed and three meal contexts: “at home” versus “away from home,” “alone” versus “with company,” and “with screen time” versus “without screen time.” A population ratio approach was used to estimate HEI-2015 total and component scores for all food items consumed by meal context. Mean HEI-2015 scores (range: 0–100) for the three meal context variables were compared using t-tests. Where there were significant differences in total scores, additional t-tests were used to explore which HEI-2015 components were the primary drivers. All tests were stratified by sex and adjusted for multiple comparisons.

**Results:**

HEI-2015 scores were lower for meals consumed away vs. at home (mean difference (SE), males: − 8.23 (1.02); females: − 7.29 (0.93); both *p* < 0.0001) and for meals eaten with vs. without company (mean difference (SE), males: − 6.61 (1.06); females: − 7.34 (1.18); both *p* < 0.0001). There was no difference comparing with vs. without screen time. When HEI-2015 component scores were examined, fewer total fruits, whole grains, and dairy were consumed away from home or with company; more total vegetables and greens and beans, and less added sugars were consumed with company.

**Conclusions:**

Our findings suggest an association between the behavior cues of meal location and companions and dietary choices among older adults. Future studies can explore the individual and interactive effects of meal context on diet quality and subsequent health outcomes.

## Background

The U.S. Department of Agriculture (USDA) Healthy Eating Index 2015 (HEI-2015) is a widely accepted dietary index that assesses the extent one adheres to the 2015–2020 Dietary Guidelines for Americans [1]; a higher score indicates higher diet quality. A healthy diet is widely associated with a reduced risk for chronic disease and mortality, including diabetes, cardiovascular disease, and various cancers [2–5]. However, on average, older adults in the U.S. do not meet dietary recommendations. In a nationally representative survey, participants 18–64 and ≥ 65 years of age on average scored 58 and 66 out of 100 on the HEI-2015, respectively [6]. Given the ample room for improvement in diet quality, identifying behavioral strategies to promote healthier diets may benefit efforts to improve population health.

Few studies in older adults have examined the impact of meal contexts on eating behaviors and diet quality: what one eats may be strongly influenced by where, with whom, and with what surroundings one eats. In the U.S, meals consumed away versus at home tend to include larger portion sizes and more energy-dense, low-quality foods/beverages [7], although, the preferences and decisions of the primary home meal provider also affect diet quality [8]. Social support, the communication it involves, and behavioral modeling from meal companions may also influence dietary choices via changes in knowledge, feelings of belonging, or self-esteem [9]; studies have shown both positive [9–11] and negative impacts of these influences on diet quality [8, 12]. Lack of social support (i.e., eating alone) has also been associated with a lower diet quality and variety, possibly driven by feelings of social isolation or a reduction in meal enjoyment [10, 13]. Lastly, studies indicate that the presence of technology, such as television, during mealtime impacts the diet quality of older adults via increased exposure to food advertisements, which may stimulate the desire for unhealthy foods and subsequent overeating regardless of hunger [14, 15]. A better understanding of the role of meal contexts as potential mediators of dietary behavior and diet quality in older adults is needed to design behavioral interventions based on psychosocial theory for this population.

Using HEI-2015 total scores, the main aim of this study was to compare the dietary quality of meals by contexts consisting of location (eating away from home versus at home), the presence of others (eating with company versus alone), and the use of electronic screens (eating with versus without the presence of electronic screens) in the Interactive Diet and Activity Tracking in AARP (IDATA) study. The second aim was to examine which components of the HEI-2015 drove differences in HEI-2015 total scores by meal context.

## Methods

### Study participants and design

The IDATA study was a measurement error study that compared diet and physical activity instruments to reference biomarkers [16]. The study was approved by the Special Studies Institutional Review Board of the National Cancer Institute (NCI). The study included a convenience sample of men and women aged 50 to 74 years old on the AARP mailing list residing in and around Pittsburgh, Pennsylvania. Of the 71,000 adults contacted by mail, 4967 visited the IDATA study website, completed registration, and were interested in telephone screening; 3515 were screened via telephone. Of the sample who completed the telephone screening, 1163 adults were further screened in-person at the IDATA study center and 1130 provided informed consent. Participants were included in the study if they understood English, had no health conditions affecting metabolism, were reasonably mobile, had reliable internet access, were not on a weight loss diet, and had no prior formal nutrition training. Of the 1130 adults who provided informed consent, 1110 adults (98%) attended study center visits and provided data.

Participants were divided into four subgroups that completed three study center visits and the same battery of assessments over a 12-month period (2012–2013), though the timing of data collection events differed by study group to optimize study center throughput. Details regarding the IDATA study protocol are described elsewhere [16, 17]. Demographic information, including sex, age, and race/ethnicity, was collected from participants during the telephone screening. Anthropometric measures, including height, weight, and waist circumference, were measured by trained personnel at each study center visit; body mass index (BMI, kg/m^2^) and waist circumference (WC, cm) were estimated by taking the mean of BMI and WC measures across the three clinic visits.

### Diet and meal context assessment

IDATA participants were tasked with completing six administrations of the 2011 version of the internet-based Automated Self-Administered 24-Hour (ASA24®) Dietary Assessment Tool (version 2011). Participants were prompted by email and robotic telephone call to complete a self-administered ASA24 on a random day at bimonthly intervals for 12 months (i.e., during months 1, 3, 5, 7, 9, and 11, for a total of up to 6 recalls). Second and third reminder emails and robotic calls were sent to participants as needed.

The ASA24 provides a list of foods, drinks, and supplements and employs multiple prompts for respondents to provide details about portion size, actual amount eaten, and cooking style to aid recall of everything consumed from midnight to midnight the preceding day [17, 18]. Participants first chose one of six meal types (breakfast, brunch, lunch, dinner/supper, snack, or drink were considered meals in this analysis) and reported the time of day it was consumed. They were then asked to report each meal’s context - operationalized as meal location, concurrent use of a television or computer, and presence of others. Participants were asked, “Where did you eat this food?”, and response options included “Home,” “Fast food restaurant,” “Other restaurant,” “Cafeteria,” “Bar or tavern,” Work (not in cafeteria),” “Car,” “Sports or entertainment venue,” “Someplace else,” and “Don’t know.” When asked, “Were you watching TV and/or using the computer while eating this meal?”, participants responded, “Watching TV,” “Using a computer,” “Watching TV and using a computer,” “Neither of these,” or “Don’t know.” Participants were then asked, “Did you eat with anyone?”, to which they responded “Yes,” “No,” or “Don’t know”; for those who selected “Yes,” they then indicated if they ate with “Family member(s),” “Other(s),” “Family member(s) and Other(s),” or “Don’t know.” Lastly, participants reported in detail the food, drink(s), and/or supplement(s) consumed.

Participants who completed at least one ASA24 recall were included in the analysis (number of recalls: ≥1: *n* = 1021; ≥2: *n* = 992; ≥3: *n* = 957; ≥4: *n* = 884; ≥5: *n* = 833; ≥6: *n* = 679). Responses to the three meal contexts were collapsed into at home versus away from home (“Someplace else” was collapsed into away from home; “Don’t know” was collapsed into at home), with screen time versus without screen time (“Don’t know” was collapsed into without screen time), and alone versus with company (“Don’t know” was collapsed into alone), respectively. Nutrient and food group intake estimates were calculated using the USDA’s Food and Nutrient Database for Dietary Studies (FNDDS) version 4.1 [19], MyPyramid Equivalents Database, version 2.0 [20], and the National Health and Nutrition Examination Survey (NHANES) Dietary Supplement Database 2007–2008 [21].

### Characterizing quality of foods consumed in different meal contexts

The Healthy Eating Index 2015 (HEI-2015) scores diet quality using 13 components based on the key recommendations in the 2015–2020 Dietary Guidelines for Americans: total fruits, whole fruits, total vegetables, greens and beans, whole grains, dairy, total protein foods, seafood and plant proteins, fatty acids, refined grains, sodium, added sugars, and saturated fats [1, 22]. The latter four components are scored as moderation components, whereby greater consumption results in lower sub-scores.

Throughout this analysis, the population ratio approach was adapted to characterize the HEI total and component scores for all food items consumed in a particular meal context [23]. The construction of each component for a meal context is illustrated for the total fruit HEI component and the eating at home context:

$$ \mathrm{Total}\ \mathrm{fruit}\ \mathrm{HEI}\ {\mathrm{component}}_{\mathrm{at}\ \mathrm{home}}=\mathrm{Total}\ {\mathrm{fruit}\ \mathrm{servings}}_{\mathrm{at}\ \mathrm{home}}/{\mathrm{Total}\ \mathrm{energy}}_{\mathrm{at}\ \mathrm{home},} $$

where the numerator and denominator are computed by summing fruit servings and energy (kcal) for all food items eaten at home. The HEI scoring algorithm is applied to this ratio to calculate the HEI component score for total fruits eaten at home. This process is applied to all 13 HEI components for all six meal context conditions. The HEI component scores were summed to produce the total HEI score for each meal context, ranging from 0 to 100, with a higher score indicating higher quality. Thus, the HEI-2015 components and scores were calculated for foods within a context of consumption, not for participants.

### Statistical analysis

Because participants could contribute different amounts of information (ranging from one to six recalls) to the numerator and denominator of an HEI component by meal context, the standard errors of total HEI score, component scores, and differences between scores by context were estimated using a jackknife resampling procedure, operating at the individual level [24]. That is, the HEI components for all contexts were re-computed sequentially after leaving out all recalls from one person at a time, and the variability of those estimates was used to estimate standard errors. This approach accounted for both the fact that recalls from a given person are related, as well as the fact that a single person’s recalls can contribute to multiple HEI component estimates.

To compare HEI profiles across meal contexts, a gatekeeping procedure was used as follows. The mean total HEI scores were compared using t-tests for all meals for each of the three meal context variables by sex. A Bonferroni adjustment was used to account for multiple comparisons; *p* < 0.0083 (0.05/6) was considered statistically significant. For any meal context comparison where there was a significant difference in total score, additional t-tests were conducted to examine which of the 13 HEI components might be the driving differences. For the combined set of these additional t-tests, adjustment for multiple comparisons was performed using the Benjamini-Hochberg procedure, with the nominal false discovery rate (FDR) set at 2.5% [25].

All analyses were stratified by sex. Descriptive characteristics were calculated as mean values and standard deviations unless otherwise indicated.

## Results

Of the 1110 participants who consented and provided data, 89 did not complete at least one ASA24; therefore, the analysis included 1021 participants, evenly divided by sex (Table 1). Participants were primarily non-Hispanic white with a mean age for males and females of 64 and 62 years, respectively. Mean BMI was 28 kg/m^2^ for both males and females, and males had a higher mean waist circumference.

**Table 1:** Participant characteristics from the IDATA Study^a^

Variables	Males	Females
Sex *(N (%))*	510 (50.0)	511 (50.0)
Age (years) *(Mean (SD), Range)*	63.9 (5.8), 50.0–74.0	62.2 (6.1), 50.0–74.0
Race/ethnicity *(N (%))*		
Non-Hispanic White	485 (95.1)	452 (88.5)
African American	19 (3.7)	53 (10.4)
Other	6 (1.2)	6 (1.2)
BMI (kg/m^2^) *(Mean (SD))*	28.3 (4.2)	27.7 (4.9)
Waist circumference (in) (Mean (SD))	39.0 (4.4)	35.2 (4.7)

^a^ *IDATA* Interactive Diet and Activity Tracking in AARP, *HEI-2015* 2015 Healthy Eating Index, *SD* standard deviation

When comparing scores for all meals by meal context (Table 2), total HEI scores for meals eaten away from home were significantly lower than meals eaten at home for both males and females. Total HEI-scores for meals eaten with company were also significantly lower than meals eaten alone for both males and females. There was no significant difference in total HEI-scores for meals in the context of screen time (Table 2).

**Table 2:** Comparison of total HEI-2015 scores by meal context and sex in the IDATA Study (*N* = 1021)^a^

	Meal location (Mean (SE))			Presence of others (Mean (SE))			Presence of a screen^b^ (Mean (SE))		
	Away from home	At home	Difference	With company	Alone	Difference	With screen time	Without screen time	Difference
**Males**	56.78 (0.88)	65.00 (0.74)	−8.23 (1.02)^c^	59.34 (0.77)	65.95 (0.88)	−6.61 (1.06)^c^	61.88 (0.90)	63.44 (0.67)	−1.57 (0.98)
**Females**	62.53 (0.77)	69.83 (0.79)	−7.29 (0.93)^c^	62.15 (0.77)	69.49 (1.00)	−7.34 (1.18)^c^	67.00 (1.03)	67.40 (0.64)	−0.40 (1.02)

^a^HEI-2015, Healthy Eating Index-2015; *IDATA* Interactive Diet and Activity Tracking in AARP

^b^Defined as concurrently watching television and/or using the computer

^c^*p* < 0.0001

Differences in HEI-2015 component scores were analyzed by meal location and the presence of others, stratified by sex. Two meal contexts for 13 HEI component scores required 26 possible comparisons for both males and females, for a total of 52 possible comparisons. For nine subcomponents, the estimated differences and standard errors were zero. The remaining 43 t-tests were performed using the Benjamini-Hochberg procedure, and 27 differences were judged statistically significant. Among men, meals eaten away from home had lower mean scores for total fruits, whole grains, dairy, and refined grains and higher mean scores for whole fruits and fatty acids compared to meals eaten at home; findings were similar among females, excluding whole fruits for which the difference was not statistically significant (Table 3). Among men, meals eaten with company had lower mean scores for total fruits, whole fruits, whole grains, dairy, sodium, and saturated fats, and higher mean scores for total vegetables, greens and beans, and added sugars when compared to meals eaten alone; findings were similar among females, excluding whole fruits and saturated fats (Table 4).

**Table 3:** Comparison of HEI-2015 component scores by meal location and sex in the IDATA Study (*N* = 1021)^a^

		Males (*n* = 510)			Females (*n* = 511)		
HEI-2015 components	Maximum points	Away from home (Mean (SE))	At home (Mean (SE))	Difference (Mean (SE))	Away from home (Mean (SE))	At home (Mean (SE))	Difference (Mean (SE))
Total fruits	5	2.54 (0.16)	4.02 (0.15)	−1.48 (0.20)^b^	3.59 (0.17)	4.72 (0.16)	−1.13 (0.23)^b^
Whole fruits	5	4.07 (0.30)	5.00 (0.00)	0.93 (0.30)^b^	5.00 (0.00)	5.00 (0.00)	0.00 (0.00)
Total vegetables	5	4.05 (0.13)	4.03 (0.09)	0.02 (0.15)	4.73 (0.13)	4.84 (0.13)	−0.11 (0.17)
Greens & beans	5	3.09 (0.21)	3.23 (0.17)	−0.13 (0.26)	4.98 (0.27)	5.00 (0.16)	−0.02 (0.30)
Whole grains	10	2.19 (0.15)	4.65 (0.17)	−2.47 (0.20)^b^	2.30 (0.14)	4.67 (0.15)	−2.38 (0.20)^b^
Dairy	10	4.35 (0.15)	6.60 (0.17)	−2.25 (0.22)^b^	5.30 (0.17)	6.73 (0.18)	−1.43 (0.24)^b^
Total protein foods	5	5.00 (0.00)	5.00 (0.00)	0.00 (0.00)	5.00 (0.00)	5.00 (0.00)	0.00 (0.00)
Seafood & plant proteins	5	5.00 (0.00)	5.00 (0.00)	0.00 (0.00)	5.00 (0.00)	5.00 (0.00)	0.00 (0.00)
Fatty acids	10	4.70 (0.19)	3.66 (0.16)	1.04 (0.24)^b^	4.62 (0.27)	3.87 (0.24)	0.75 (0.26)^b^
Refined grains	10	6.80 (0.22)	7.50 (0.19)	−0.70 (0.27)^b^	6.96 (0.23)	8.56 (0.19)	−1.60 (0.27)^b^
Sodium	10	2.29 (0.24)	2.80 (0.17)	−0.51 (0.27)	2.69 (0.23)	3.33 (0.20)	−0.63 (0.27)
Added sugars	10	8.02 (0.19)	8.24 (0.14)	−0.22 (0.19)	7.95 (0.16)	8.08 (0.13)	−0.13 (0.18)
Saturated fats	10	4.66 (0.20)	5.27 (0.18)	−0.61 (0.25)	4.42 (0.28)	5.03 (0.24)	−0.61 (0.26)

^a^ Healthy Eating Index-2015; *IDATA* Interactive Diet and Activity Tracking in AARP, HEI-2015, SE, standard error

^b^ The Benjamini-Hochberg method determined this comparison to be statistically significant with a false discovery rate of 2.5%

**Table 4:** Comparison of HEI-2015 component scores by presence of others and sex in the IDATA Study (*N* = 1021)^a^

		Males (*n* = 510)			Females (*n* = 511)		
HEI-2015 components	Maximum points	With company (Mean (SE))	Alone (Mean (SE))	Difference (Mean (SE))	With company (Mean (SE))	Alone (Mean (SE))	Difference (Mean (SE))
Total fruits	5	2.57 (0.12)	5.00 (0.00)	−2.43 (1.21)^b^	2.89 (0.13)	5.00 (0.00)	−2.11 (0.13)^b^
Whole fruits	5	3.69 (0.20)	5.00 (0.00)	−1.31 (0.20)^b^	4.45 (0.24)	5.00 (0.00)	−0.55 (0.23)
Total vegetables	5	5.00 (0.00)	2.73 (0.22)	2.27 (0.13)^b^	5.00 (0.00)	3.81 (0.14)	1.19 (0.14)^b^
Greens & beans	5	3.81 (0.17)	2.21 (0.22)	1.60 (0.27)^b^	5.00 (0.00)	3.86 (0.25)	1.14 (0.25)^b^
Whole grains	10	3.12 (0.16)	5.33 (0.23)	−2.21 (0.26)^b^	2.85 (0.13)	5.07 (0.19)	−2.22 (0.22)^b^
Dairy	10	5.52 (0.15)	6.78 (0.22)	−1.26 (0.23)^b^	5.75 (0.19)	6.92 (0.20)	−1.17 (0.25)^b^
Total protein foods	5	5.00 (0.00)	5.00 (0.00)	0.00 (0.00)	5.00 (0.00)	5.00 (0.00)	0.00 (0.00)
Seafood & plant proteins	5	5.00 (0.00)	5.00 (0.00)	0.00 (0.00)	5.00 (0.00)	5.00 (0.00)	0.00 (0.00)
Fatty acids	10	3.72 (0.16)	4.16 (0.23)	−0.44 (0.27)	3.91 (0.20)	4.05 (0.40)	−0.14 (0.44)
Refined grains	10	7.30 (0.19)	7.27 (0.25)	0.03 (0.30)	7.78 (0.22)	8.45 (0.23)	−0.67 (0.29)
Sodium	10	1.22 (0.19)	4.53 (0.27)	−3.31 (0.32)^b^	1.22 (0.22)	4.81 (0.24)	−3.59 (0.32)^b^
Added sugars	10	8.89 (0.14)	7.17 (0.20)	1.71 (0.21)^b^	9.00 (0.13)	7.29 (0.16)	1.71 (0.18)^2^
Saturated fats	10	4.51 (0.18)	5.77 (0.23)	−1.26 (0.27)^b^	4.29 (0.23)	5.22 (0.37)	−0.93 (0.42)

^a^ HEI-2015, Healthy Eating Index-2015; IDATA, Interactive Diet and Activity Tracking in AARP; SE, standard error

^b^ The Benjamini-Hochberg method determined this comparison to be statistically significant with a false discovery rate of 2.5%

## Discussion

The primary purpose of this study was to compare the quality of foods consumed by older adults in different meal contexts. Though meal context may play a mediating role and impact the effectiveness of dietary behavior change interventions, there is a limited understanding to date of the association between meal context and diet quality in this population. Meals consumed away from home or with company had lower total HEI-2015 scores compared to meals consumed at home or alone, respectively. No statistically significant difference was found in total HEI-2015 scores when meals were consumed with and without concurrent use of television or computer screens. Secondarily, when HEI-2015 component scores were examined by meal context, total fruits, whole grains, and dairy were consumed less when meals were either eaten away from home or with company, and more total vegetables and greens and beans and less added sugars were consumed when meals were eaten with company.

Our results complement those of past studies. A 2010 report by the USDA Economic Research Service found that each meal adults ate away from home continuously increased their total daily calories and lowered their HEI-2005 scores as well as lowered whole grain and whole fruit consumption [26]. A 2011 systematic review also found eating away from home was associated with greater total energy and fat intake and lower intake of many micronutrients in U.S. adults, and fast foods were the main type of foods consumed away from home [7]. When Kirkpatrick et al. assigned HEI-2005 scores to menus from popular U.S. fast food restaurants, total scores were indeed generally poor; menus scored especially poor on whole fruits, whole grains, dark green and orange vegetables and legumes, and energy from fats and sugars [27].

Our findings also suggest an association between the behavior cues of meal companions and dietary choices. For example, older adults’ diets may be shaped by the diets and social support of peers or living partner(s) (e.g., spouse, adult children), particularly by the household members who prepare meals [8]. A review by Cruwys et al. [28] included 69 experimental modeling studies that manipulated the eating behavior of a social referent and measured food choice or intake. Participants reported that food choices and intake were shaped by the norms that others provided. Modeling occurred because participants factored in appropriate peer behaviors and a sense of belonging into their food choices. Thus, if social norms and judgements motivate older adults to alter typical eating behaviors to fit those of dining companions [29] and those behavioral cues *are not* supportive of healthy eating, it would be unsurprising for diet quality to worsen with company compared to eating alone. Conversely, if the behavioral cues of eating companions *are* supportive of healthy eating and there is a variety of foods prepared and consumed by companions, diet quality may improve. This may explain why we found that more total vegetables and beans and greens were consumed when meals were eaten with company compared to those eaten alone. Another explanation may be the positive impact of social support. Eating alone may be simply due to a busy schedule that limits time for meal preparation [30] or due to being single, widowed, or socially isolated with a small social network. Both scenarios may lower motivation to cook complex meals and have been shown in various older adult populations to negatively impact diet variety and quality [10, 11, 13, 30].

In contrast to other studies of older adults, no difference in the diet quality of meals was found when comparing meals with versus without the presence of a television or computer screen. One study by Sisson et al. [15] found that diet quality was inversely associated with television viewing, with those in the lowest HEI quartile more likely to watch > 4 h of television daily compared to those in the highest quartile. Huffman et al. [14] similarly examined this association using 24-h diet recalls (24HRs) in a nationally representative sample of youth, adolescents, and adults 19 years of age and older in NHANES. Though screen time was inversely associated with diet quality across all age groups, they did not investigate driving components within the total HEI-2005 score. Additionally, they did not examine diet quality by meal type (i.e., they looked at HEI scores for the entire day) or stratify by age group (i.e., separating older adults from middle-aged and younger adults), either of which may explain the differences from our findings. Another explanation may be a potential shortcoming of our study, given that the ASA24 2011 version did not inquire about phones, tablets, or other screens when querying screen time during a meal. The omission of smart devices existing in 2011 as part of screen time may thus have impacted our findings.

Overall, our findings suggest that it is important for future behavioral interventions, public health nutrition messaging, and education to not only provide dietary guidance to older adults on how to consume high-quality diets, but to also consider the influence of the meal context on meal preferences and expected results. Specifically, the ability to adhere to a healthy diet or long-term dietary changes may be mediated by where and with whom individuals usually eat. These contexts may have more impact with aging due to potential changes in lifestyle, including widowhood or a smaller social network.

### Strengths and limitations

A major strength of this study was the use of multiple 24HRs via the ASA24 to collect dietary data. Unlike a food frequency questionnaire which requires participants to recall dietary data over the past months or year, the ASA24 only asks participants to recall food consumed over the past 24 h, reducing the risk of recall bias [31]. Additionally, our study looked not only at overall diet quality through the HEI-2015, but also examined the HEI-2015 components driving the index’s relationship with the meal contexts.

As with any study utilizing self-report dietary assessment measures, the findings in this study are influenced by measurement error. Despite the challenges inherent in dietary assessment, investigations like the IDATA Study are still needed to characterize dietary behaviors for public health intervention. Limitations specific to the IDATA Study include a lack of population representation, as the study relied on dietary recalls from a convenience sample of older adults with little racial and ethnic diversity. Additionally, we did not examine potential interactions among the three meal context variables collected by the ASA24. A study in Canadian adults found that eating alone versus with others impacted diet quality for meals eaten at home, but not away from home [9], suggesting an interaction between meal location and the presence of others. A similar interaction may occur between screen time and the presence of others, whereby the former may negate the latter [15, 32]. Our study focused on studying main effects of meal contexts and did not expand further to explore interactions due to the large growth of multiple comparisons it would entail and our limited sample size. A future larger study in older adults can build on our findings and examine the interactions of meal contexts. Lastly, as previously mentioned, our study did not ask about the use of cell phones, tablets, or other screens that may be present during a meal.

## Conclusions

Our findings suggest that meal location and the presence of others are contextual factors that are significantly associated with diet quality in older adults. Future longitudinal studies and interventions in diverse populations are needed to further explore the individual and interactive effects meal contexts may have on older adults’ diet quality and subsequent health outcomes. Such research may be especially important in older populations given the changes in social relationships with aging and the rapid changes occurring in the food environments in which we live.

## Data Availability

The dataset for the ASA24 instrument analyzed in the current study are available in the iDATA repository, https://prevention.cancer.gov/research-groups/biometry/interactive-diet-and-activity.

## Abbreviations

24HR: 24-h recall
ASA24: Automated Self-Administered 24-Hour (ASA24®) Dietary Assessment Tool
BMI: Body Mass Index
FDR: False Discovery Rate
FNDDS: Food and Nutrient Database for Dietary Studies
HEI-2005: Healthy Eating Index-2005
HEI-2015: Healthy Eating Index-2015
IDATA: Interactive Diet and Activity Tracking in AARP
NCI: National Cancer Institute
SD: Standard deviation
SE: Standard error
USDA: U.S. Department of Agriculture
WC: Waist Circumference
